# Remarkable functions of *sn*-3 hydroxy and phosphocholine groups in 1,2-diacyl-*sn*-glycerolipids to induce clockwise (+)-helicity around the 1,2-diacyl moiety: Evidence from conformation analysis by ^1^H NMR spectroscopy

**DOI:** 10.3762/bjoc.13.196

**Published:** 2017-09-25

**Authors:** Yoshihiro Nishida, Mengfei Yuan, Kazuo Fukuda, Kaito Fujisawa, Hirofumi Dohi, Hirotaka Uzawa

**Affiliations:** 1 Nanobiology Course in Graduate School of Advanced Integration Science & Molecular Chirality Research Center, Chiba University, Matsudo 271-8510, Chiba, Japan; 2 Nanomaterials Research Institute, National Institute of Advanced Industrial Science and Technology (AIST), 1-1-1 Higashi, Tsukuba 305-8565, Japan

**Keywords:** cell membrane, chirality, conformation, deuterium labeling, *sn*-glycerol, glycerolipids, glycerophospholipids, helicity, Karplus equation, proton NMR spectroscopy, staggered conformers

## Abstract

Cell-membrane glycerolipids exhibit a common structural backbone of asymmetric 1,2-diacyl-*sn*-glycerol bearing polar head groups in the *sn*-3 position. In this study, the possible effects of *sn*-3 head groups on the helical conformational property around the 1,2-diacyl moiety in the solution state were examined. ^1^H NMR Karplus relation studies were carried out using a series of 1,2-dipalmitoyl-*sn*-glycerols bearing different *sn*-3 substituents (namely palmitoyl, benzyl, hydrogen, and phosphates). The ^1^H NMR analysis indicated that the helical property around the 1,2-diacyl moiety is considerably affected by these *sn*-3 substituents. The *sn*-3 hydroxy group induced a unique helical property, which was considerably dependent on the solvents used. In CDCl_3_ solution, three staggered conformers, namely gt(+), gg(−) and tg, were randomized, while in more polar solvents, the gt(+) conformer with (+)-helicity was amplified at the expense of gg(−) and tg conformers. The *sn*-3 phosphocholine in phosphatidylcholine exhibited a greater effect on the gt(+) conformer, which was independent of the solvents used. From the ^1^H NMR analysis, the helical conformational properties around the 1,2-diacyl moiety conformed to a simple empirical rule, which permitted the proposal of a conformational diagram for 1,2-dipalmitoyl-*sn*-glycerols in the solution states.

## Introduction

Glycerophospholipids, constituting the basic elements of cytoplasm bilayer membranes, are responsible for several cell functions [1–3]. These chiral biomolecules have an asymmetric *sn*-glycerol backbone. Although *sn*-glycerol is symmetric, an *sn*-3 phosphate group makes it chiral with an (*R*)-configuration at the *sn*-2 position [4]. Such molecular chirality is crucial to not only their biological activities but also for their metaphysical properties, as glycerophospholipids comprise elements of fluid membrane [5] and nanoscale vesicles called liposomes [6].

**Figure 1:**
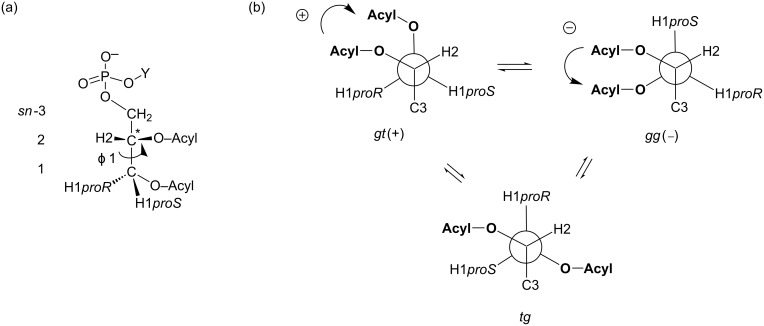
(a) Structures of cell-membrane glycerophospholpids with a common asymmetric 1,2-diacyl-*sn*-glycerol-3-phosphate structure and (b) the conformational equilibrium among three staggered conformers, namely gt(+), gg(−) and tg around the 1,2-diacyl moiety.

In addition, the chiral *sn*-glycerol backbone is composed of acyclic polyols that produce several conformers through the free rotation about each of the C–C single bonds. For example, the free rotation about the *sn*-1,2 and *sn*-2,3 C–C bonds furnishes nine conformers by the combination of three staggered rotamers, namely gt (*gauche*–*trans*), gg (*gauche–gauche*) and tg (*trans–gauche*, Figure 1). Conformational flexibility often leads to the ambiguous characterization of acyclic molecules, thereby making it difficult to precisely examine their biological activities. This observation is applicable for cell-membrane glycerophospholipids that have been targets in numerous conformational studies [7–15].

Cell-membrane glycerophospholipids are known to adopt the gt(+) and gg(−) conformations around the 1,2-diacyl moiety (Figure 1). From X-ray crystallography data, a common structure in which the 1,2-diacyl chains are aligned in parallel is observed, which adopts either the gt(+) or gg(−) conformer [7,10,12]. An analogous conformation has been reportedly observed among α-glycosyl 1,2-diacyl-*sn*-glycerols in the solution state [16]. Probably, the two *gauche* conformers, namely gt(+) and gg(−), are stabilized in a manner so as to permit stacking interactions between the 1,2-diacyl chains.

In our previously reported circular dichroism (CD) studies [17–18], helical conformational properties of a series of 1,2-dibenzoyl-*sn*-glycerols bearing different *sn*-3 substituting groups were examined. As shown in Figure 1, gt(+) is one of the *gauche* conformers with a right-handed (+)-helicity around 1,2-diol, while gg(−) is another *gauche* conformer with an antipodal left-handed (−)-helicity. Harada and Nakanishi [19] reported the dibenzoate chirality CD methodology, which helps in the analysis of the chirality originating from the disparity between these two helical conformers. We have found thereby that the 1,2-dibenzoyl moiety favors the right-hand screwed gt(+) conformer over the left-handed one [17]. The gt(+)-preference was kept irrespective of the *sn*-3 substituting groups and the solvents used. Moreover, a relation in the order as gt(+) > gg(−) > tg was maintained. On the other hand, the intensity of exciton couplet CD bands changed remarkably among the 1,2-dibenzoyl-*sn*-glycerols [18], indicating that the disparity between gt(+) and gg(−) conformers varies widely by influences from *sn*-3 groups.

**Scheme 1:**
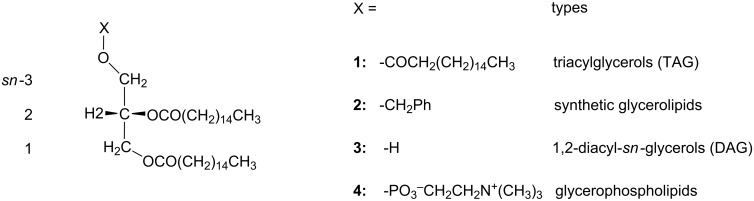
The four 1,2-dipalmitoyl-*sn*-glycerols **1–4** examined in this study.

Helical properties constitute one of the major factors in determining the molecular chirality [20] of not only proteins and nucleic acids but also simpler biomolecules [17–19] such as acyclic *sn*-glycerols and glycerophospholipids [8,21]. In this study, the helical properties of four 1,2-dipalmitoyl-*sn*-glycerols **1**−**4** (Scheme 1) are examined; these 1,2-dipalmitoyl-*sn*-glycerols are composed of different substituents (X) at the *sn*-3 position, and each of them serves as a representative model for the 1,2-diacyl-*sn*-glycerols, as categorized in Scheme 1. Although the exciton chirality CD methodology is not applicable for these 1,2-diacyl-*sn*-glycerolipids without an appropriate UV/CD chromophore, ^1^H NMR spectroscopy will permit the precise determination of their helical conformational properties.

## Results and Discussion

### 1. Helical conformational properties of tripalmitin **1** and 3-*O*-benzyl 1,2-dipalmitoyl-*sn*-glycerol (**2**) in CDCl_3_ solutions

First, the helical property of tripalmitin **1** (entry 1, Table 1) is examined according to a previously reported method [18]. Briefly, fractional populations (%) of the three staggered conformers [gt(+), gg(−) and tg] are calculated using two Karplus equations, Equation 1 [22] and Equation 2 [18]. From the conformer populations (%), the “helicity index” is determined according to the method previously reported by our group [18].

[1]
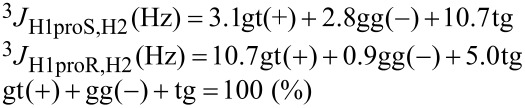


[2]
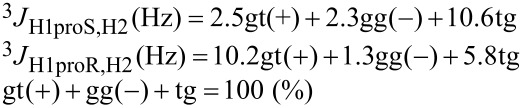


**Table 1:** ^1^H NMR data and helical conformational properties of tripalmitin **1** and 3-*O*-benzyl derivative **2** in the solution state.

Entry	Compound (head X = )	Solvent^a^	^1^H NMR data δ (ppm) ^3^*J* (Hz)		Populations (%) of staggered conformers in *sn*-1,2 position						Helicity index in *sn*-1,2 position		

					Equation 1			Equation 2			Equation 2 (Equation 1)		
					
			H1*proR*	H1*proS*	gt(+)	gg(−)	tg	gt(+)	gg(−)	tg	Sign (+/−)	Disparity [gt−gg]%	Volume [gt+gg]%
			
1	**1**^b^ (palmitoyl)	CDCl_3_	4.15 6.0	4.29 4.4	44	37	19	41	35	24	+	6 (7)	76 (81)
2	**2** (-CH_2_Ph)	CDCl_3_	4.19 6.4	4.34 3.8	52	37	11	49	34	17	+	15 (15)	83 (89)
3		C/M (10:1)	4.19 6.5	4.34 3.8	53	36	11	50	33	17	+	17(17)	83 (89)

^a^C/M (v/v) represents the ratios of the mixed solvents CDCl_3_ (C) and methanol-*d*_4_ (M). ^b^Discrimination between H*_proR_* and H*_proS_* as well as the acquisition of their ^1^H NMR data are carried out according to our previously reported studies [23–24] and in the Materials and methods section of this paper.

The result in entry 1 (Table 1) indicates that tripalmitin **1** favors gt(+) with right-handed (+)-helicity compared to gg(−) with left-handed helicity (helical disparity = +6%−7%). According to our previously reported study [18], the disparity, as estimated from Equation 2, is linear with respect to the magnitude and intensity of exciton coupling CD bands, indicating that the 1,2-diacyl moiety in **1** exhibits (+)-chirality corresponding to the equilibrium imbalance between gt(+) and gg(−) conformers as indicated by the helicity index (entry 1 in Table 1). The helical volume of **1** (76% by Equation 2 and 81% by Equation 1) indicates that this glycerolipid favors the two helical conformers in addition to the antiperiplanar tg conformer (ca. 25% by Equation 2) at equilibrium.

Next, the helical property of chiral 3-*O*-benzyl derivative **2** is examined. In our previously reported CD study [17], the intensity of the exciton couplet CD bands for 3-*O*-benzyl-1,2-dibenzoyl-*sn*-glycerol is greater than those of 3-palmitoyl-1,2-dibenzoyl-*sn*-glycerol. From the preceding result, the replacement of the *sn*-3 palmitoyl group in **1** with a benzyl ether is expected to enhance the helical property. As can be seen from the result of **2** (Table 1, entries 2 and 3), the helical disparity (+15%, Equation 1 and Equation 2) increases with the introduction of a benzyl group. This result is in good agreement with our expectation. In addition, the helical volume (%) was increased by 7–8% as compared with that of **1**. The 3-*O*-benzyl group apparently enhances the (+)-chirality around the 1,2-diacyl moiety.

To examine the possible effects of solvents, the helical property of **2** is also examined in a mixed solvent containing ca. 10% methanol-*d*_4_ in CDCl_3_ (C/M 10:1, v/v). The result in entry 3 (Table 1) indicates that the helical property of **2** is marginally affected by protic solvents.

### 2. Helical conformational property of chiral 1,2-dipalmitoyl-*sn*-glycerol (**3**) using different solvents

Next, the helical property of 1,2-dipalmitin **3** with a hydroxy (OH) group in the *sn*-3 position is examined. This compound is selected as a representative model of 1,2-diacyl-*sn*-glycerols, which play essential roles in the metabolism and anabolism of glycerolipids [25–28]. Compound **3** is prepared by the catalytic hydrogenolysis of benzyl ether **2** (for the synthetic details, see Supporting Information File 1).

**Figure 2:**
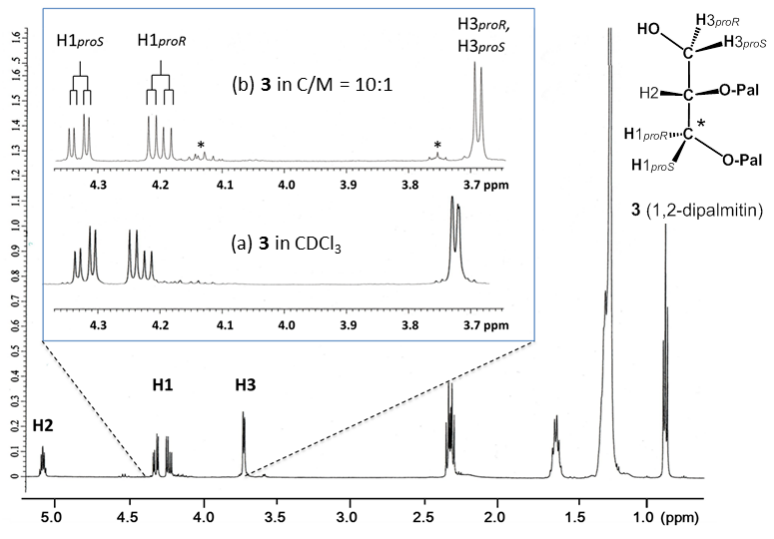
^1^H NMR spectra of 1,2-dipalmitin (**3**) in CDCl_3_ after partial isomerization into the 1,3-isomer. (a) The expanded spectrum of **3** in CDCl_3,_ (b) **3** in a mixed solvent with ca. 10% methanol-*d*_4_ in CDCl_3_ (C/M ca 10:1, v/v). The signal marked with an asterisk * corressponds to a 1,3-diacyl isomer, which is derived from **3** during storage in a CDCl_3_ solution.

The ^1^H NMR spectrum of **3** in a CDCl_3_ solution (Figure 2a) shows a pair of double doublet signals of H1*proS* (δ 4.32 ppm) and H1*proR* (δ 4.23 ppm), which exhibit a spectral feature similar to that of **1** [23]. On the other hand, the signals of H3*proR* and H3*proS* in **3** collapse in a narrow region around δ 3.73 ppm. These observations are in good agreement with the ^1^H NMR data of **3** reported by Vilceze and Bittman [29].

From the analysis of the ^1^H NMR data using Equations 1 and 2, 1,2-dipalmitin **3** in CDCl_3_ exhibits a very unique helical conformational property. That is, the populations of the gt(+) and gg(−) conformers are almost equal to give a helical disparity of around 0% (Table 2, entries 1 and 2). A helical volume of around 75% (Equation 2) is analogous to that observed in **1**. In contrast to the ^1^H NMR data of **2**, those of **3** showed remarkable changes in the “mixed solvents” containing methanol-*d*_4_ in CDCl_3_. With the addition of methanol-*d*_4_, the H1*proR* and H1*proS* signals shift to high and low fields, respectively (Figure 2b). Simultaneously, the H3 signals shift upfield by 0.04 ppm. The shift of these H1 signals increases with an increase in the content of methanol-*d*_4_ in the mixed solvents, while the H3 signals are marginally changed; thereafter, their positions are maintained at δ 3.69 ppm (Figure 2b). As shown in Table 2, entries 1–6, the change in the chemical shifts is related to that in the vicinal coupling constants, indicative of a change in the dynamic conformations occurring around the 1,2-diacyl moiety in **3**.

**Table 2:** ^1^H NMR data and helical conformational properties of 1,2-dipalmitin **3** using different solvents.

Entry	Compound (head X = )	Solvent^a^	^1^H NMR data δ (ppm) ^3^*J* (Hz)		Populations (%) of staggered conformers in *sn*-1,2 position						Helicity index in *sn*-1,2 position		

					Equation 1			Equation 2			Equation 2 (Equation 1)		
					
			H1*proR*	H1*proS*	gt(+)	gg(−)	tg	gt(+)	gg(−)	tg	Sign (+/−)	Disparity [gt−gg]%	Volume [gt+gg]%
			
1	**3** (-H)	CDCl_3_	4.23^b^ 5.6	4.33^b^ 4.5	40	40	20	35	39	26	–	−4 (0)	74 (80)
2		CDCl_3_	4.23 5.7	4.32 4.4	41	40	19	37	39	24	–/+	−2 (1)	76 (81)
3		C/M (10:1)	4.20 6.2	4.33 4.0	48	38	13	45	35	20	+	10 (10)	80 (86)
4		C/M (5:1)	4.19 6.4	4.34 3.7	52	38	9	49	35	16	+	14 (14)	84 (90)
5		C/M (2:1)	4.19 6.5	4.37 3.7	53	37	10	50	34	16	+	16 (16)	84 (90)
6		C/M (2:1) + D_2_O	4.18 6.6	4.37 3.5	55	38	7	53	34	13	+	19 (17)	87 (93)

^a^C/M (v/v) represents the ratios of the mixed solvents CDCl_3_ (C) and methanol-*d*_4_ (M). ^b1^H NMR data from the study reported by Vilceze and Bittman [29].

From the analysis of the ^1^H NMR data using the Karplus equations (Equation 1 and 2), an equilibrium shift mainly occurs between the gt(+) and tg conformers. In the mixed solvents with high methanol-*d*_4_ contents, the population of the gt(+) conformer seemingly increases at the expense of the tg conformer. The population of the gg(−) conformer decreases by several percent after the addition of ca. 10% of methanol-*d*_4_ (Table 2, entry 3). Thereafter, the gg(−) population remains constant at around 35% irrespective of the solvents.

**Figure 3:**
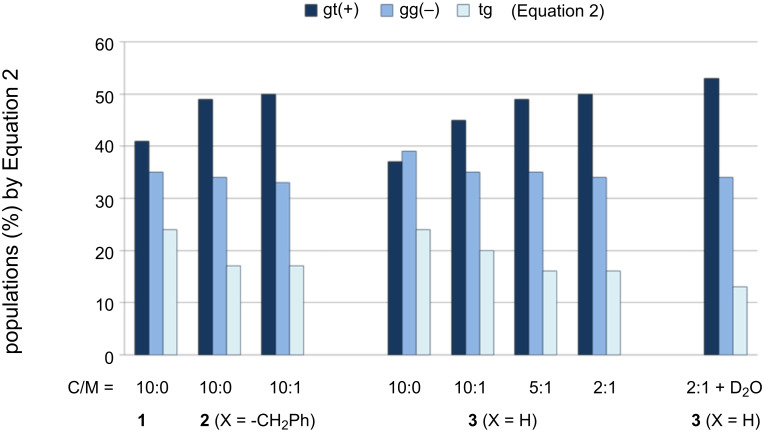
Fractional populations (%) of the three staggered conformers around the *sn*-1,2 C–C single bond in 1,2-dipalmitoyl-*sn*-glycerols **1**–**3** bearing different substituents (X) at the *sn*-3 position. Populations (%) are calculated from Equation 2 and each of the populations possibly includes deviations within ±3% by digital resolution (<0.12 Hz) of ^1^H NMR spectroscopy (500 MHz).

Because of the shift in the equilibrium from tg to gt(+) in the mixed solvents with high methanol contents, the helical disparity (%) and helical volume (%) increase. With an increase in the methanol-*d*_4_ content to 17% (C/M 5:1), the helical property of **3** becomes similar to that of **2** (Figure 3). Although this change seems to be saturated in the mixed solvent containing 33% methanol-*d*_4_ (C/M 2:1, v/v), the addition of one aliquot of D_2_O to this solution further changes the gt(+) and tg populations by a few percent (Table 2, entry 6 and Figure 3). Moreover, the H2 signal of **3** shifts downfield by 0.03 ppm in the presence of D_2_O, although this signal marginally changes in the mixed solvents without D_2_O.

From the ^1^H NMR spectra in Figure 2, a part of **3** is isomerized to 1,3-isomer during storage in solutions. To examine the possible effects from this isomer, the isomerization is promoted up to 50%, and the ^1^H NMR spectrum of the isomeric mixture is analyzed. This experiment indicates that the presence of the 1,3-isomer marginally affects the ^1^H NMR signals of **3**.

As shown in Table 1, entries 2 and 3, the solvents marginally affect the ^1^H NMR signals of **2**. Clearly, *sn*-3 OH plays an essential role in the conformational dynamics, as shown above. The dynamic change is probably caused by solvation by methanol-*d*_4_ and/or D_2_O around the 3-OH group as well as the increasing polarity of the mixed solvent. As judged from the chemical shift change in the H3 signals, the solvation is possibly saturated in the mixed solvent with 10% methanol-*d*_4_ (C/M = 10:1). In the solvent containing more than 33% methanol-*d*_4_ (C/M = 2:1), the solvation by methanol-*d*_4_ might be partly replaced with D_2_O.

Hamilton et al. [30] employed ^13^C NMR spectroscopy to examine the dynamic molecular behavior of 1,2-dilauroyl-*sn*-glycerol located in liposomes mixed with glycerophospholipids. Their ^13^C NMR analysis revealed that the hydration occurring around the carbonyl groups in the 1,2-diacyl moiety triggers the dynamics of the molecular alignments in liposomes. Probably, an analogous phenomenon related to the solvation around *sn*-3 OH was observed. Thus, solvation is thought to play a key role in the dynamic conformation change around the 1,2-diacyl moiety.

### 3. Helical conformational properties of 1,2-dipalmitoyl-*sn*-glycero-3-phosphocholine (**4**, **DPPC**) and other glycerophospholipids in the solution state

**Scheme 2:**
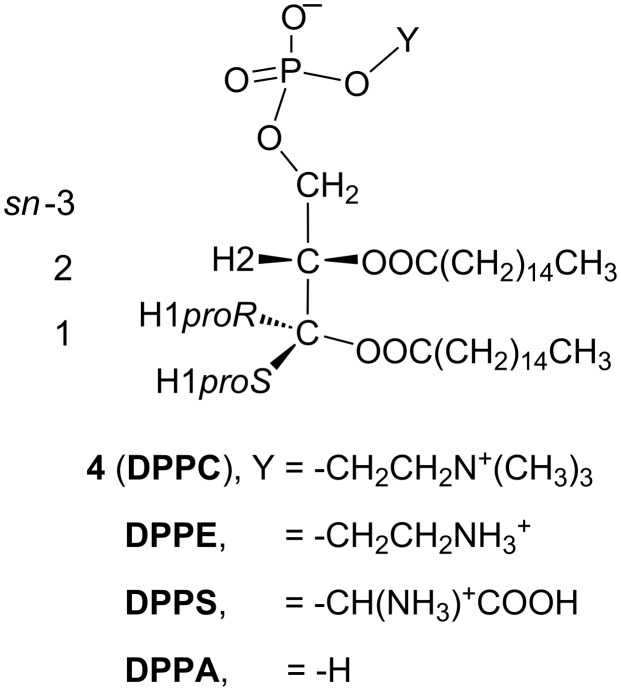
Structures of glycerophospholipids with a common structural skeleton of 1,2-dipalmitoyl-*sn*-glycerol 3-phosphate. Abbreviations: **DPPC** =1,2-dipalmitoyl-*sn*-glycero-3-phosphocholine, **DPPE** = 1,2-dipalmitoyl-*sn*-glycero-3-phosphoethanolamine, **DPPS** = 1,2-dipalmitoyl-*sn*-glycero-3-phospho-L-serine, **DPPA** = 1,2-dipalmitoyl-*sn*-glycerol 3-phosphate.

The current ^1^H NMR analysis is extended to four 1,2-dipalmitoyl-*sn*-glycerophospholipids (Scheme 2) bearing different terminal groups (Y). Large portions of their ^1^H NMR data were collated by Hauser et al. [10]. In our experiment, the ^1^H NMR data of phosphatidylcholine **4** are obtained using the mixed solvent C/M = 10:1.

**Figure 4:**
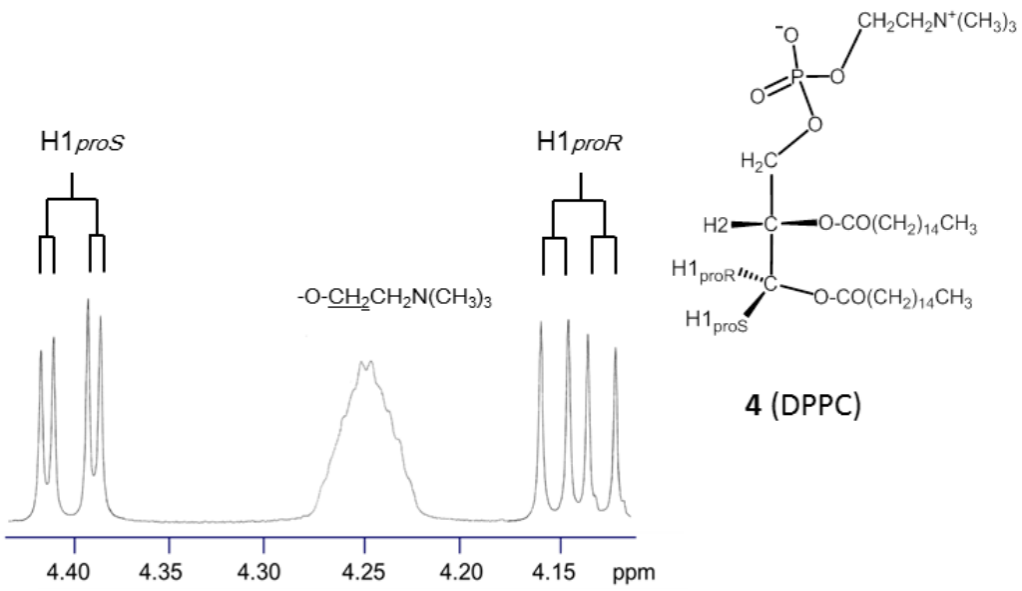
Partial ^1^H NMR spectrum of **4** in a mixture of CDCl_3_ and methanol-*d*_4_ (C/M = 10:1, v/v).

As shown in Figure 4, the ^1^H NMR spectrum of **4** shows a pair of well-separated double doublet signals of H1*proR* (δ 4.14 ppm) and H1*proS* (δ 4.40 ppm). Compared to the other 1,2-diacyl-*sn*-glycerols **1**–**3**, this phospholipid exhibits a higher vicinal coupling constant to H1*proR* (^3^*J*_H1R,H2_ = 7.2 Hz) and a lower one to H1*proS* (^3^*J*_H1S,H2_ = 3.5 Hz). In addition, the difference in the chemical shift (Δδ = 0.26 ppm) between the H1*proR* and H1*proS* signals increases in **4**. These observations predict that the 1,2-diacyl moiety in **4** exhibits an extremely unique conformational property.

In fact, the ^1^H NMR Karplus analysis indicates that the helical disparity of **4** increases above 30% (Table 3, entries 1 and 2); the disparity is greater than that observed thus far in previously reported studies [16–18]. When previously reported ^1^H NMR data for **4** are examined [8,10,31], the strong (+)-chirality is independent of the solvents used (Table 3, entries 1–4). Moreover, the data in entries 5−7 (Table 3) indicate that this property is commonly observed in the glycerophospholipids listed in Scheme 2**,** indicating that an *sn*-3 phosphate group plays a key role. From Table 3, the *sn*-3 phosphate group can also simultaneously increase the helical volume (%). The helical volumes (%) of **4** using Equation 1 nearly reach the theoretical limit (100%). This result is in good agreement with the conformational properties of cell-membrane glycerophospholipids reported previously [10–15]. On the other hand, in our calculations using Equation 2 as the advanced Karplus equation [18], the helical volumes of these glycerophospholipids are around 90%, which permits the presence of the tg conformer by ca. 10%. Note, that the tg conformer is crucial [32–33] because the antiperiplanar relation is thought to deform lamellar phases and trigger membrane fusion.

**Table 3:** ^1^H NMR data of 1,2-dipalmitoyl-*sn*-glycero-3-phospholipids and their helical conformational properties in solution states.

Entry	Compound	Solvent^a^	^1^H NMR δ (ppm) ^3^*J* (Hz)		Populations (%) of staggered conformers around *sn*-1,2						Helicity index in *sn*-1,2 position		

					Equation 1			Equtation 2			Equation 2 (Equation 1)		
					
			H1*proR*	H1*proS*	gt(+)	gg(−)	tg	gt(+)	gg(−)	tg	Sign (+/−)	Disparity [gt−gg]%	Volume [gt+gg]%
			
1	**4** (**DPPC**)	CDCl_3_	4.13^b^ 7.3	4.40^b^ 2.9	66	35	−1	64	30	6	+	34 (31)	94 (101)
2		C/M (10:1)	4.14 7.2	4.40 3.5	62	32	6	59	27	13	+	32 (30)	86 (94)
3		C/M (2:1)	4.16^c^ 6.9	4.42^c^ 3.1	61	38	1	59	33	8	+	26 (23)	92 (99)
4		CD_3_OD	4.18^d^ 7.0	4.42^d^ 3.2	61	36	3	59	31	10	+	28 (25)	91 (97)
5	**DPPE**^c^	C/M (2:1)	4.18 6.9	4.40 3.4	59	36	5	57	31	12	+	26 (23)	88 (95)
6	**DPPS**^c^	C/M (4:3)	4.19 7.2	4.43 3.0	64	36	0	63	30	7	+	33 (28)	93 (100)
7	**DPPA**^c^	C/M (2:1)	4.21 7.1	4.40 3.5	61	33	6	59	28	13	+	31 (28)	87 (94)

^a^C/M (v/v) represents the ratios of the mixed solvents CDCl_3_ (C) and methanol-*d*_4_ (M). ^b1^H NMR data obtained from a database of Spectral Database for Organic Chemistry (SDBS), No. 16108HSP-45-792 in http://sdbs.db.aist.gojp/sdbs/vgi-bin/direct_frame_top.cgi [31]. ^c1^H NMR data from a paper of Hauser et al. [10]. ^d1^H NMR data from a paper of Bruzik et al. [8].

With respect to the antiperiplanar tg conformer, Hauser et al. [10] examined the effect of self-assembly using 1,2-dihexanoyl (C6) homologs of glycerophospholipids. They added these acyl homologs into D_2_O at concentrations less than or greater than the critical micellar concentration. In their ^1^H NMR spectroscopy analysis, the tg conformer is almost absent under the self-assembled conditions [10]. In addition, in our calculation by Equation 2, the helical volume (%) reaches the theoretical limit (100%), and the helical disparity (%) is greater 40% [18]. Probably, cell-membrane glycerophospholipid **4** can adopt the unusual rotational mode, where the 1,2-diacyl chains swing between gt(+) and gg(−) conformers. However, such extraordinary rotation would be possible only when molecules are located under self-assembled conditions.

### 4. General trend in the helical conformational properties of 1,2-dipalmitoyl-*sn*-glycerols **1**–**4** in the solution state

**Figure 5:**
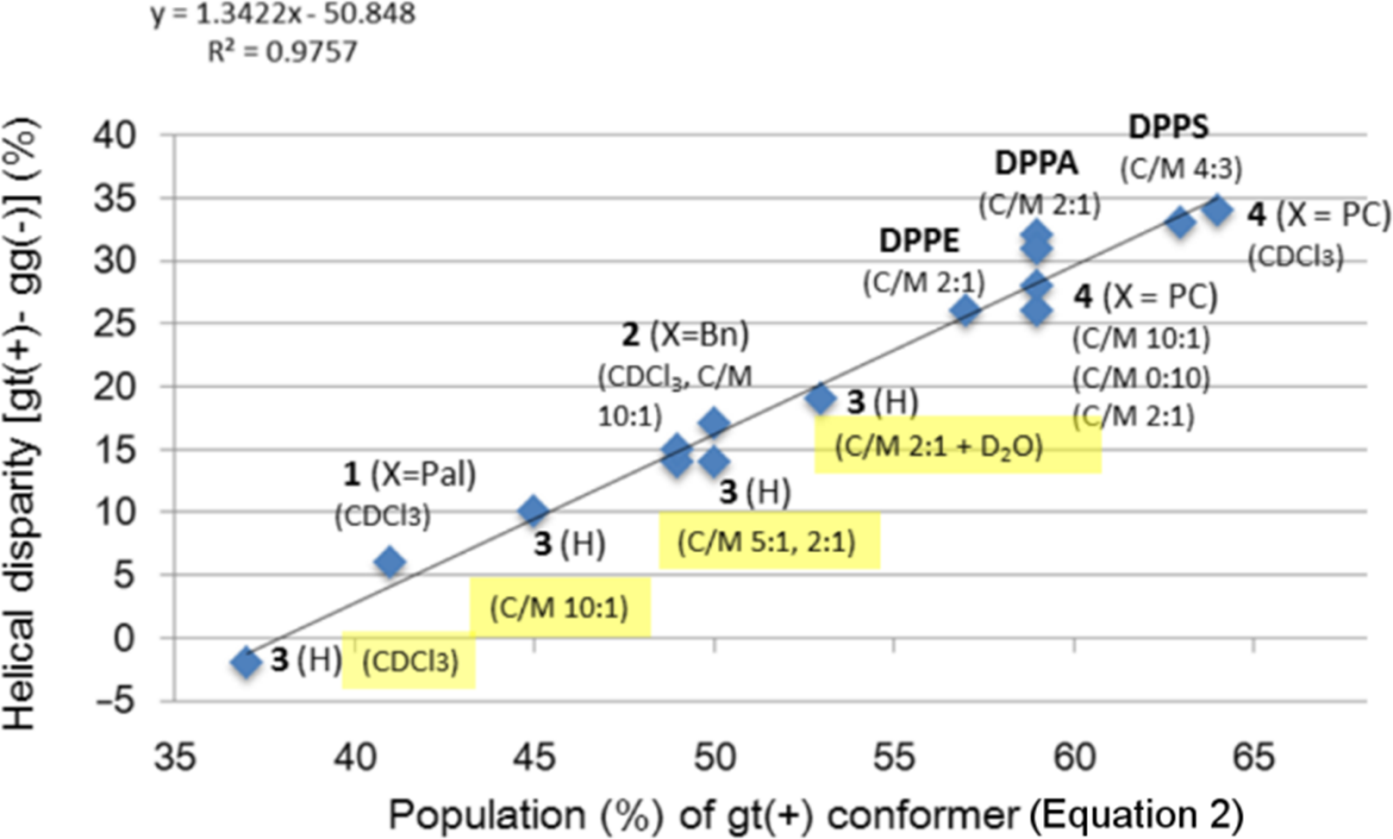
Linear relation between the helical disparity (%) and gt(+) population (%) as observed for the helical conformational properties of 1,2-dipalmitoyl-*sn*-glycerols **1**–**4** in the solution state.

By plotting the helical disparity (%) obtained by Equation 2 against the population (%) of the gt(+) conformers for glycero1ipids **1**–**4** examined herein, a linear relation (y = 1.34x − 50.8, R^2^ = 0.976) is obtained (Figure 5).

From the linearity, we obtain Equation 3 and Equation 4:

[3]
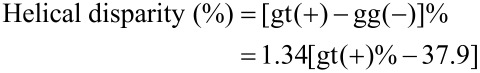


[4]
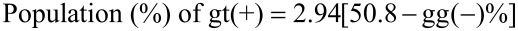


Equation 3 indicates that the helical disparity (%) increases as a function of gt(+) population (%). Equation 4 indicates that the population (%) of the gt(+) conformer increases at the expense of the gg(−) conformer. When the rule of 100 > gt(+) > 0 (%) is applied to Equation 4, the gg(−) population can assume values in a narrow range between 25% and 51%. At a gg(−) population of 25%, the gt(+) population and helical volume (%) reach their theoretical limits (75% and 100%, respectively). At a gg(−) population of 51%, the gt(+) population reaches 0% (tg = 49%).

**Figure 6:**
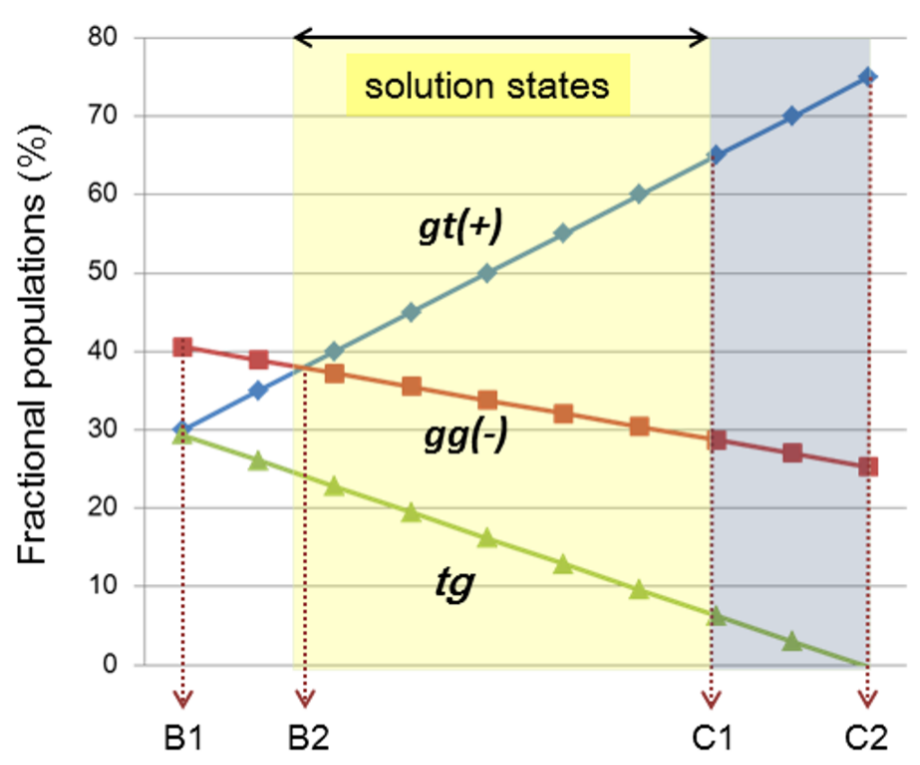
An empirical diagram showing helical conformational properties around 1,2-diacyl moiety in asymmetric 1,2-dipalmitoyl-*sn*-glycerols in solution states.

When the gt(+) population is arbitrarily changed between 30% (B1 section) and 75% (C2 section) in these empirical formulae, a diagram shown in Figure 6 is obtained. The derived diagram is apparently useful for summarizing the overall helical conformational properties of the four 1,2-dipalmitoyl-*sn*-glycerols **1**–**4**.

In this diagram, an intersection, denoted by B2, is observed, indicating that the helical disparity becomes 0% when both gt(+) and gg(−) populations are 38%. At this point, the helical volume is 76%, and the tg population is 24%. 1,2-Dipalmitin **3** exhibits a similar behavior when dissolved in CDCl_3_ (Table 2, entry 2). When methanol-*d*_4_ is added to the CDCl_3_ solution of **3**, the gt(+) population increases from 37% up to 50% at the expense of the gg(−) and tg conformers. The observed change is well reproduced in this diagram. Glycerophospholipid **4** shows the largest gt(+) population (64%) in the CDCl_3_ solution (Table 3, entry 1). A similar situation is denoted by a section C1, where the populations of gt(+), gg(−) and tg are 64%, 29% and 7%, respectively. These values are in good agreement with the experimental results (Table 3, entry 1).

In Table 4, the applicability of Equation 3 and Equation 4 is evaluated using α-D- and α-L-glucopyranosyl 1,2-dipalmitoyl-*sn*-glycerols (Table 4, entries 1–4). The helical conformational properties of these α-glycolipids are determined by Equation 2 applying the ^1^H NMR data reported in a preceding paper [16]. The results of the ^1^H NMR analyses are compared with those calculated by Equation 4. Entries 1–4 (Table 4) indicate that Equation 4 can reproduce also the helical conformational properties of these α-glycolipids.

**Table 4:** Helical conformational properties of α-D- and α-L-glucopyranosyl 1,2-dipalmitoyl-*sn*-glycerols in the solvent mixture of CDCl_3_ and methanol-*d*_4_ (C/M = 10:1).

Entry	Compound^a^ (head groups at *sn*-3)	Results^b^ (%) from ^1^H NMR spectroscopic analyses by Equation 2					Calculated values^c^ (%) with Equation 4				

		gt	gg	tg	dispariity	volume	gt	gg	tg	disparity	volume
		
1	α-D-Glc	53	36	11	17	89	53	33	14	20	86
2	6-phosphocholine α-D-Glc	53	36	11	17	89	53	33	14	20	86
3	6-palmitoyl α-D-Glc	49	37	14	12	86	49	34	17	15	83
4	6-phosphocholine α-L-Glc	55	33	12	22	88	55	32	13	23	87

^a^Abbreviations: α-D- or α-L-Glc = α-D- or α-L-glucopyranoside, ^b1^H NMR data in our preceding study [16] are analyzed with Equation 2; ^c^calculated values (%) from Equation 4 by adapting the gt population (%) in the ^1^H NMR spectroscopy analysis.

## Conclusion

In this study, a ^1^H NMR spectroscopy analysis of 1,2-dipalmitoyl-*sn*-glycerols **1**–**4** in the solution state was carried out to elucidate their helical conformational properties around the 1,2-diacyl moiety. In addition, the possible effects from the substituents at the *sn*-3 position were evaluated. In the current analysis, the chiral ^2^H-labeled triacylglycerols [23–24] provided a key basis to discriminate between the H1*proR* and H1*proS* signals (Materials and methods). Throughout this study, each of the 1,2-diplamitoyl-*sn*-glycerols **1**–**4** exhibited a unique helical property, indicating that not only *sn*-configurations but also *sn*-3 substituents govern the helical conformational property around the 1,2-diacyl moiety. The biological systems in nature effectively utilize the *sn*-3 substituents. For example, the *sn*-3 OH group in 1,2-diacyl-*sn*-glycerols is essential for the dynamic conformational behavior, which possibly plays major roles in their biological functions as transmembrane second messengers [25–30,34]. The *sn*-3 phosphocholine in phosphatidylcholine induced strong (+)-chirality regardless of the solvents used, which should considerably contribute to their functions as activators of membrane-bound glycoproteins [35–37].

The helical conformational properties observed in the four 1,2-dipalmitoyl-*sn*-glycerols (Scheme 1) conformed to an empirical rule, as shown in Equation 3 and in the diagram shown in Figure 6. This rule revealed that the helical disparity (%) linearly changes by the function of gt(+) populations, albeit in an allowed range. Probably, the range between B2 and C1 sections in the diagram covers the conformational properties of most 1,2-diacyl-*sn*-glyceols in the solution state. The conformational properties in this region can be characterized by the relation of gt(+) > gg(−) > tg (%), which has been commonly observed in our preceding studies [16–18].

The ^1^H NMR spectroscopy analysis was carried out in organic solvents. It is possible that the conclusions obtained herein deviate from those examined under physiological conditions. For example, glycerophospholipids are located in self-assembled lamellar structures that show liquid crystalline properties. Plasma membranes comprise glycerophospholipids which interact with other membrane components such as glycoproteins and sterols [38–39]. Moreover, natural glycerolipids are composed of heterogeneous acyl chains with different alkyl lengths and alkenyl –C=C– bonds. Thus, it will be of high significance in extensional studies to evaluate the helical conformational properties of 1,2-diacyl-*sn*-glycerols assuming these heterogeneous situations which may occur in nature.

## Materials and Methods

### Model compounds

Tripalmitin **1** was prepared together with chirally deuterated *sn*-glycerols and identified in our former studies [22–23]. 1,2-Dipalmitoyl-*sn*-glycerol (**3**) and its 3-*O*-benzyl derivative **2** were prepared in a reported manner [8,29] (for details, see Supporting Information File 1). 1,2-Dipalmitoyl-*sn*-glycero-3-phosphocholine (**4 DPPC**) was purchased from Tokyo Kasei Co. Ltd. and used without purification. All the compounds studied here have chemical purities over 95% (^1^H NMR) except for **3** which isomerizes into the 1,3-diacyl isomer during storage in CDCl_3_ solution.

#### Acquisition of the ^1^H NMR spectral data of H1*proR* and H1*proS* signals

Each of the four glycerolipids **1**–**4** is dissolved in either CDCl_3_ or the mixed solvents containing methanol-*d*_4_ in CDCl_3_ (deuterium content > 99.5%) at ca. 10 mM concentrations. ^1^H NMR spectroscopy is measured on a JEOL 400 MHz or 500 MHz instruments at temperatures between 22–25 °C. Chemical shifts (δ, ppm) and coupling constants (^3^*J*, Hz) of H1*proR* and H1*proS* signals are obtained manually with ^1^H NMR spectra expanded in the region between δ 4.0 ppm and δ 4.5 ppm. The manual process is of high significance for the current ^1^H NMR analysis since a peak top by computer system does not always point at a weighted center correctly.

**Scheme 3:**
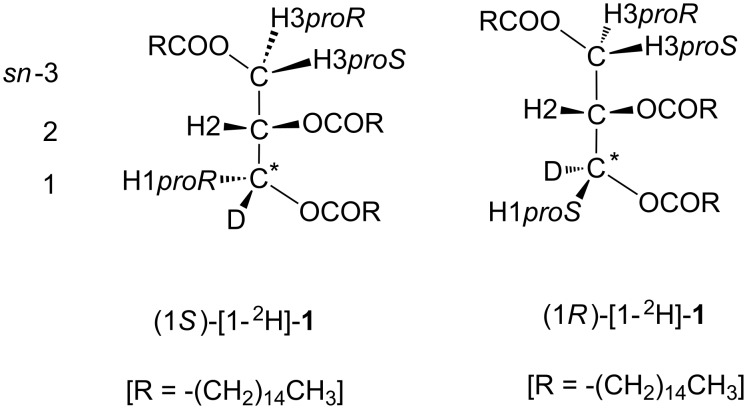
Chirally ^2^H-labelled tripalmitins (1*S*)- and (1*R*)-1-[^2^H]-**1** [23].

The discrimination between H1*proR* and H1*proS* signals is another crucial process. In our former studies [22–23], chiral ^2^H-labelled triacylglycerols were prepared (Scheme 3) and applied for the assignment of these diastereomeric protons, namely H1*proR* and H1*proS*. The results have shown an empirical relation between the two H1 signals; the H1*proS* signals appear downfield from the H1*proR* signals (δ H1*proS* > δ H1*proR* ppm) and have lower smaller coupling constants (^3^*J*_H1_*_proR_*_,H2_ > ^3^*J*_H1_*_proS_*_,H2_ Hz). This rule is maintained among 1,2-diacetyl-, 1,2-dipalmitoyl-, and 1,2-dibenzoyl-*sn*-glycerols and substituents at the *sn*-3 position. The validity of this rule is confirmed in a comparative analysis using circular dichroism (CD) spectroscopy [17–18]. The current study applies these relations established in our preceding ^1^H NMR and CD studies.

#### Calculation of fractional populations (%) of three staggered conformers around the 1,2-diacyl group with a Karplus relation

A general Karplus equation of Haasnoot et al. [40] is extended into the simultaneous linear equations Equation 1 [22] and Equation 2 [18].

From the vicinal coupling constants (^3^*J* Hz) of H1*proR* and H1*proS* signals, the fractional populations (%) of the three staggered conformers are calculated. Equation 1 is a standard equation, in which the three staggered conformers have the dihedral angles of ± 60° or 180° around 1,2-diols.

Equation 2 is an advanced equation [18], which is optimized for the analysis of 1,2-diacyl-*sn*-glycerols in the solution state. The results by Equation 1 and Equation 2 produce some deviations each other. In general, Equation 1 tends to overestimate the population (%) of gt(+) and gg(−) conformers by 3–5% compared to those by Equation 2. The current study applies both Equation 1 and Equation 2 in parallel while the main discussion utilizes the results by Equation 2 as the advanced equation.

#### Definition of ‘helicity index’, ‘helical disparity (%)’ and ‘helical volume (%)’

The ‘helicity index’ [18] comprises three items, namely ‘(+) or (−)-sign’, ‘helical disparity (%)’ and ‘helical volume (%)’. The helical disparity (%) is the difference in populations (%) between gt(+) and gg(−) conformers. The disparity has either a ‘(+) or (−)-sign’, which corresponds to the sign of exciton couplet CD bands. When the gt(+) conformer is preferred over the gg(−) conformer, the sign is positive. The absolute value in the helical disparity (%) corresponds to the magnitude of the exciton couplet CD bands.

The helical volume (%) is the summation of gt(+) and gg(−) conformers. The volume expresses to what extent a given glycerolipid can adopt the two helical conformers around the 1,2-diacyl moiety. The helical volume (%) may reach the theoretical limit (100%) under self-assembled conditions [18].

## Supporting Information

File 1 Experimental and copies of spectra.
