# Supplementary material for: Remarkable functions of sn-3 hydroxy and phosphocholine groups in 1,2-diacyl-sn-glycerolipids to induce clockwise (+)-helicity around the 1,2-diacyl moiety: Evidence from conformation analysis by 1H NMR spectroscopy
Source: Beilstein J Org Chem. 2017 Sep 25;13:1999–2009. doi: 10.3762/bjoc.13.196 (PMC5629397; doi:10.3762/bjoc.13.196)
Supplement: File 1 — Experimental and copies of spectra. [file Beilstein_J_Org_Chem-13-1999-s001.pdf]

Supporting Information

for

Remarkable functions of *sn*-3 hydroxy and phosphocholine groups in 1,2-diacyl-*sn*-glycerolipids to induce clockwise (+)-helicity around the 1,2-diacyl moiety: Evidence from conformation analysis by ^{1}H NMR spectroscopy

Yoshihiro Nishida*[1], Mengfei Yuan[1], Kazuo Fukuda[1], Kaito Fujisawa[1], Hirofumi Dohi[1] and Hirotaka Uzawa[2]

Address: [1]Nanobiology Course in Graduate School of Advanced Integration Science & Molecular Chirality Research Center, Chiba University, Matsudo 271-8510, Chiba, Japan and [2]Nanomaterials Research Institute, National Institute of Advanced Industrial Science and Technology (AIST), 1-1-1 Higashi, Tsukuba 305-8565, Japan

*Corresponding author: Yoshihiro Nishida - YNishida@faculty.chiba-u.jp

Experimental and copies of spectra

Experimental

General procedures: All reactions were carried out under a dry argon atmosphere. Yields refer to chromatographically and spectroscopically homogeneous material. Anhydrous solvents were purchased from Wako Pure Chemical Industries Co., Ltd. All other chemicals for syntheses were purchased from Wako Pure Chemical Industries Co., Ltd. (Osaka, Japan), Sigma-Aldrich Co., or Tokyo Kasei Kogyo (Tokyo, Japan). All the reagents were purchased at the highest commercial quality and used without purification. All reactions were monitored by thin-layer chromatography (TLC) carried out on silica gel 60-F_{254} precoated plates (Merck) using UV light and cerium sulfate/molybdic acid solution as visualizing agent. Silica gel BW-80S (average particle size 100 mesh, Fuji Silicia Chemical Co., Ltd.) or BW-300 (average particle size 60 mesh, Fuji Silicia Chemical Co., Ltd.) was employed for flash column chromatography.

NMR spectra were recorded with a JEOL ECA 500 spectrometer equipped with Delta 5.02. Unless otherwise stated, ^{1}H NMR (500 MHz) spectra were recorded at 25 °C using internal tetramethylsilane (TMS) at 0 ppm. The following abbreviations are used to explain the multiplicities: s = singlet, d = doublet, dd = double-doublet, m = multiplet, br = broad.

***3-O-Benzyl-1,2-di-O-hexadecanoyl-sn-glycerol* (2)**: To a stirred solution of (*S*)-glycidol (5 g, 67.5 mmol) and benzyl chloride (13 g) in DMF (40 mL) cooled at 0 °C was added a powder of NaH (2.4 g) slowly for 10 minutes. The mixture was stirred at 0 °C for 1 h and then at room temperature for 3 h. After the reaction vessel was cooled to 0 °C, the mixture was treated carefully with droplets of methanol (2 mL) and stirred for 2 h at room temperature. The mixture was poured into aqueous sat. NaCl solution (100 mL) and extracted three times with a mixture of ethyl acetate and toluene (1:10, 60 mL). The extracts were combined, washed with NaCl aq. solution three times, dried over $MgSO_4$, and evaporated under diminished pressure to give **I** [(*R*)-3-*O*-benzyl-glycidol, CAS:14618-80-5] as a crude syrup (11 g), which was used in the following reaction without purification.

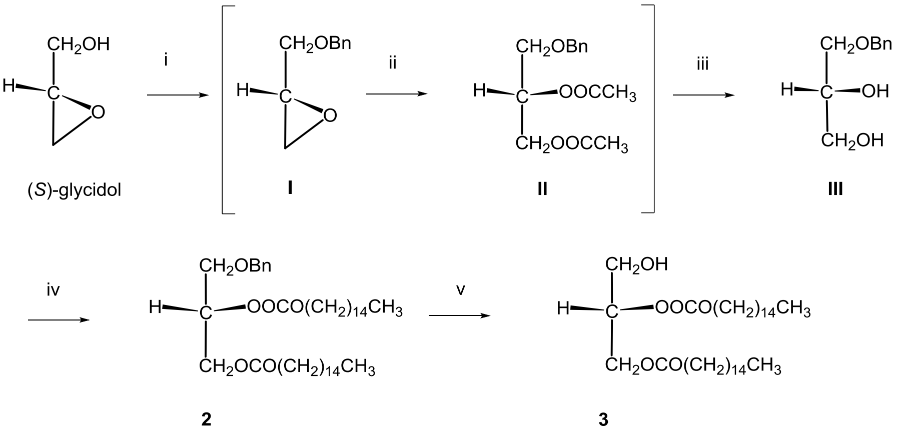

Scheme 1S

i) Benzyl chloride (1.5 mol. equiv), NaH (1.5 mol. equiv) in DMF, 0 °C~20 °C, ii) 5% CF_3COOH in Ac_2O, 0 → 30 °C, iii) K_2CO_3 in methanol, rt, iv) palmitoyl chloride (3 mol. equiv), 4-(dimethylamino)pyridine in a mixture of pyridine and CH_2Cl_2,(1/10, v/v), rt, (82% yield from (*S*)-glycidol, 4 steps), v) H_2, $Pd(OH)_2$ in a mixture of *n*-hexane and 2-propanol (1:4, v/v), rt, 96%,

The crude syrup (5 g) was dissolved in acetic anhydride (30 mL), stirred for 30 min at 0 °C, and then treated with CF_3COOH (1.5 mL) under stirring. The solution was stirred at 40 °C for 6 h. The mixture was mixed with ethanol (5 mL), stirred for 4 h, and evaporated under diminished pressure together with a mixture of ethanol and toluene to afford 1,2-diacetyl-*sn*-glycerol 3-benzyl ether **II** as syrup (8.2 g).

The syrup of **II** (5 g) was dissolved in dry methanol (50 mL), treated with anhydrous K_2CO_3 (50 mg), and stirred at room temperature for 6 h. The reaction solution was neutralized with Amberlyst-R-15 (H^+) (Aldrich), filtered, and evaporated to give **III** as syrup (3.4 g). A part of the syrup (0.5 g) was purified by column chromatography over SiO_2 with a mixture of $CHCl_3$ and methanol as eluents in a gradient mode from 1000:1 to 10/1 (v/v) to give **III** (306 mg). The ^1H NMR spectrum of **III** matched with the reported data of (*R*)-(+)-3-benzyloxyl-1,2-propanediol (CAS: 13071-59-5) reported in literature [1-3]. ^1H NMR (500 MHz, D_2O); δ_H 3.3~3.8 (4H, H1 + H3), 3.89 (1H, m, H2), 4.52 (1H, d, -CH_2Ph), 4.85 (1H, d, -CH_2Ph), 7.36 (5H, m, -PhH_5).

The purified syrup of **III** (250 mg, 1.37 mmol) was dissolved in dry pyridine (15 mL) containing 4-(dimetylamino)pyridine (50 mg). To the stirred solution was added a CH_2Cl_2 solution (5 mL) of palmitoyl chloride (942 mg, 2.5 × 1.37 mmol), and the

reaction mixture was stirred at room temperature until the reaction completed (2–6 h). The mixture was treated with sat. $NaHCO_3$ aq. solution (1 mL), stirred at room temperature for 3 h, and evaporated. The residue was diluted with a 10:1 (v/v) mixture of toluene and ethyl acetate (60 mL), and the organic layer was washed with aqueous solutions of sat. NaCl, sat. $NaHCO_3$, and again sat. NaCl. After dried over MgSO4, the organic layer was evaporated to afford a way solid (912 mg). A part of the crude solid (500 mg) was recrystallized from hot ethanol and hexane, and the purified product (378 mg) was identified as 3-*O*-benzyl-1,2-dipalmitoyl-*sn*-glycerol (**2**) (CAS: 30403-51-1) [4,5] by ^{1}H NMR spectroscopy (Figures S1 and Figure S2) in comparison with ^{1}H NMR data in a literature [5].

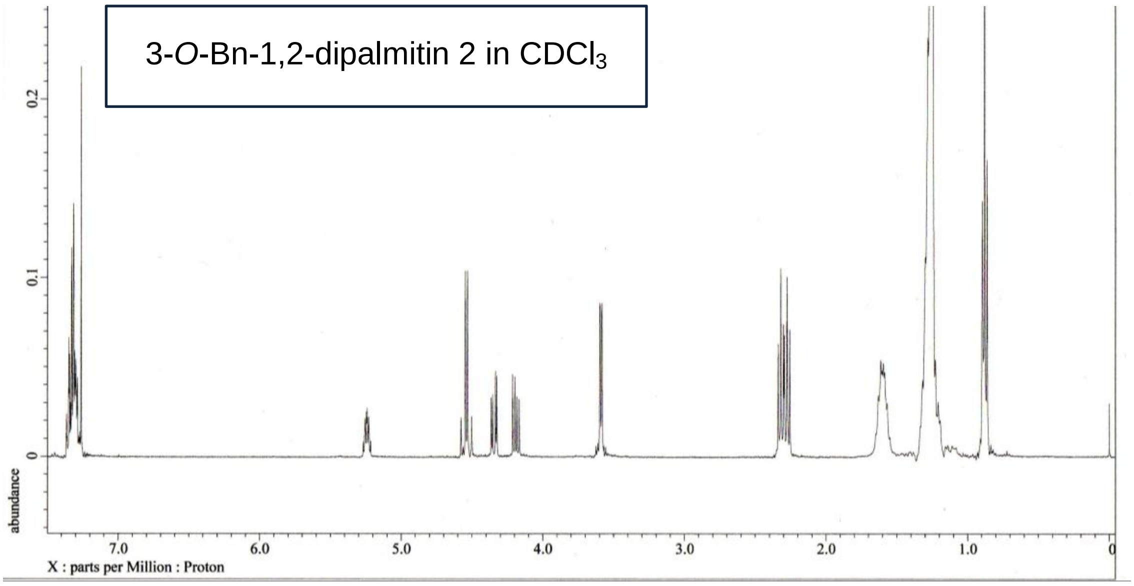

Figure S1: ^{1}H NMR spectrum of 3-*O*-benzyl-1,2-dipalmitoyl-*sn*-glycerol (**2**) in $CDCl_3$ (400 MHz).

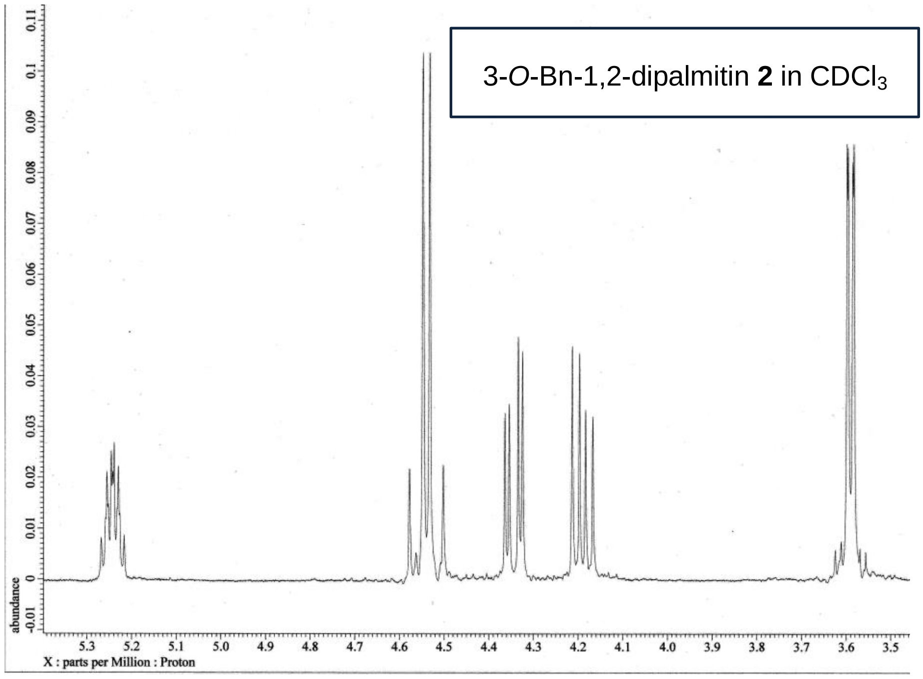

Figure S2: A partial ^{1}H NMR spectrum of 3-*O*-benzyl-1,2-dipalmitoyl-*sn*-glycerol (**2**) in $CDCl_3$ (400 MHz).

1,2-Dipalmitoyl-sn-glycerol **(3)**: The preceding product **2** (300 mg) was dissolved in a mixture of *n*-hexane and 2-propanol (1:5, 60 mL) and hydrogenated for 3 h with $Pd(OH)_2$ under a H_2 atmosphere at room temperature. After the reaction completed, the mixture was filtrated through a pad of celite eluting with a mixture of $CHCl_3$ and methanol (100:1) and evaporated to give 1,2-dipalmitoyl-*sn*-glycerol (**3,** 248 mg, 96%) as colorless powder. A part of this product (100 mg) was recrystallized from hot ethanol and used for the ^{1}H NMR spectroscopic analysis to identify the structure of **3** (CAS: 30334-71-5) (Figures S3 and Figure S4) in comparison with ^{1}H NMR data reported literature [6,7].

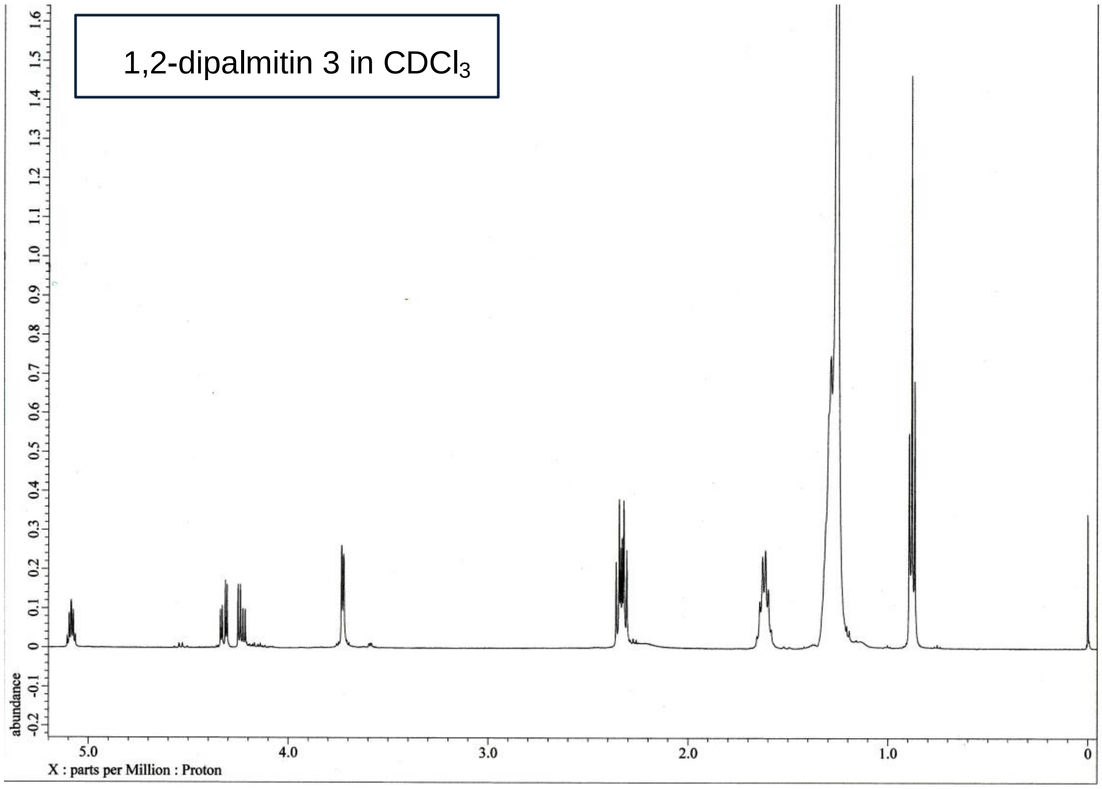

Figure S3: ^{1}H NMR spectrum of 1,2-dipalmitoyl-*sn*-glycerol (**3**) in $CDCl_3$ (500 MHz)

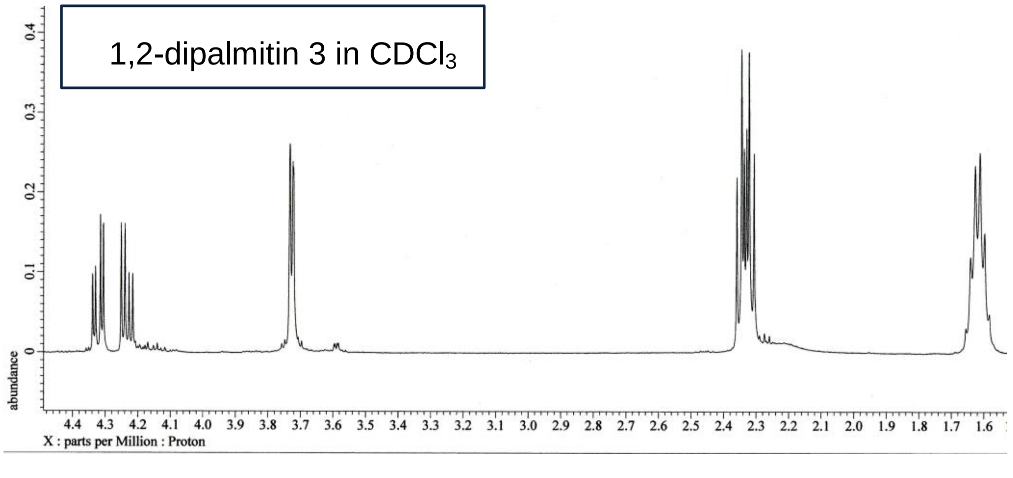

Figure S4: A partial ^{1}H NMR spectrum of 1,2-dipalmitoyl-*sn*-glycerol (**3**) in $CDCl_3$ (500 MHz)

^{1}H NMR spectroscopic measurement of 1,2-dipalmitoyl-sn-glycero-3-phosphocholine (DPPC, 4)

The titled glycerophospholipid **4** (DPPC) was purchased from Tokyo Kasei Tokyo Kasei Co. Ltd. and used without purification. This glycerophospholipid was dried under diminished pressure and dissolved in the mixed solvent containing ca. 9% methanol-d_4 in $CDCl_3$ (C/M = 10:1, v/v) to make a solution of **4** (ca. 10 mM) for analyses by 500 MHz ^{1}H NMR spectroscopy (Figure S5) .

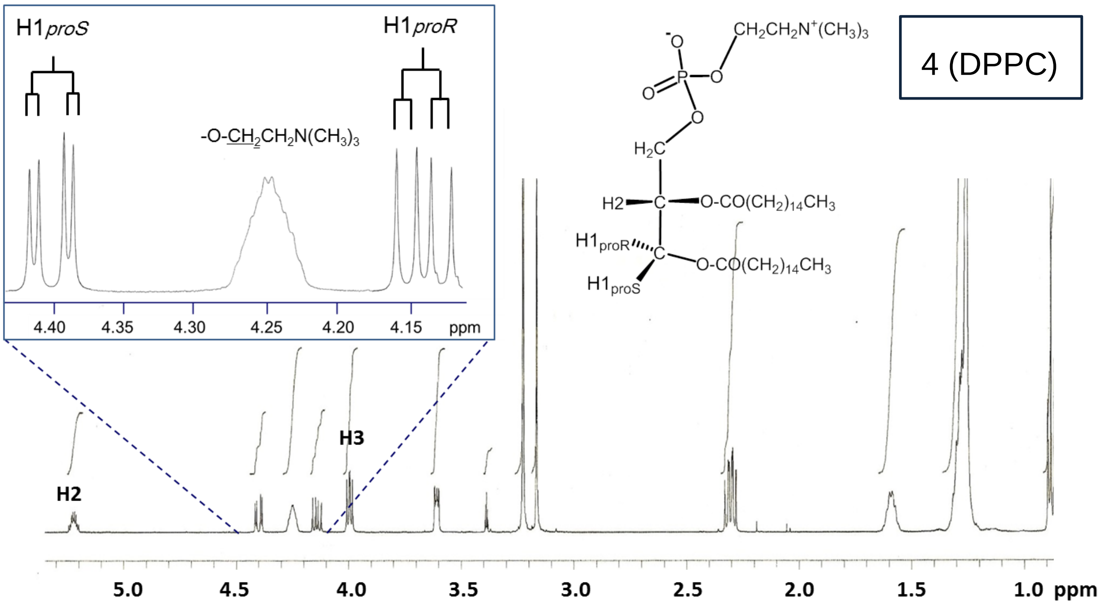

Figure S5: ^{1}H NMR spectrum (500 MHz) of **4** in a mixture of $CDCl_3$ and CD_3OD (10:1, v/v)

References:

1. Golding, B. T., and P. V. Ioannou, "Rapid syntheses of 3-*O*-benzyl-sn-glycerol and 2-*O*-benzylglycerol", *Synthesis*. **1977**. 423-424.

2. Nishida, Y.; Uzawa, H.; Hanada, S.; Ohrui, H.; Meguro, H. *Agric. Biol. Chem.* **1989**, *53*, 2319-2323.

3. Database supplied by the University of Puget Sound, Seattle (USA): http://www2.ups.edu/courses/organicchemlab/c250lab.10/HKRResults.htm.

4. Guanti, G.; Banfi, L.; Basso, A.; Bevilacqua, E.; Bondanza, L.; Riva, R. *Tetrahedron Asymmetry,* , **2004**, *15 (18)*, 2889-2892.

5. Martin, S. F.; Josey, J. A.; Wong, Y. -L.; Dean, D. W., *J. Org. Chem.*, **1994**, 59, 4805-4820.

6. Vilcheze, C.; Bittman, R. *J. Lipid Res.* **1994**, *35*, 734-738.

7. Rodriguez, J. A,; Mendoza, L. D; Pezzotti, F. ; Vanthuyne, N.; Leclaire, J.; Verger, R.; Buono, G.; Carriere, F.; Fotiadu, F., *Anal. Biochem.*, **2008**, 375 (2) 196-208.
